# A genotype × environment experiment reveals contrasting response strategies to drought between populations of a keystone species (*Artemisia tridentata*; Asteraceae)

**DOI:** 10.1002/pei3.10119

**Published:** 2023-07-24

**Authors:** Anthony E. Melton, Kara Moran, Peggy Martinez, Paige Ellestad, Erin Milliken, Walker Morales, Andrew W. Child, Bryce A. Richardson, Marcelo Serpe, Stephen J. Novak, Sven Buerki

**Affiliations:** ^1^ Department of Biological Sciences Boise State University Boise Idaho USA; ^2^ Research Computing and Data Services University of Idaho Moscow Idaho USA; ^3^ USDA Forest Service Rocky Mountain Research Station Moscow Idaho USA

**Keywords:** *Artemisia tridentata*, differential gene expression, drought response, epigenetics, keystone species, local adaptation, megadrought, sagebrush

## Abstract

Western North America has been experiencing persistent drought exacerbated by climate change for over two decades. This extreme climate event is a clear threat to native plant communities. *Artemisia tridentata* is a keystone shrub species in western North America and is threatened by climate change, urbanization, and wildfire. A drought Genotype × Environment (G × E) experiment was conducted to assess phenotypic plasticity and differential gene expression in *A. tridentata*. The G × E experiment was performed on diploid *A. tridentata* seedlings from two populations (one from Idaho, USA and one from Utah, USA), which experience differing levels of drought stress during the summer months. Photosynthetic data, leaf temperature, and gene expression levels were compared between treatments and populations. The Utah population maintained higher photosynthetic rates and photosynthetic efficiency than the Idaho population under drought stress. The Utah population also exhibited far greater transcriptional plasticity than the Idaho population and expressed genes of response pathways distinct from those of the Idaho population. Populations of *A. tridentata* differ greatly in their drought response pathways, likely due to differences in response pathways that have evolved under distinct climatic regimes. Epigenetic processes likely contribute to the observed differences between the populations.

## Graphical abstract

Big sagebrush (*Artemisia tridentata*) is a keystone species of western North America but has experienced a sharp population decline in recent decades due to human activity. A drought genotype‐by‐environment experiment was performed to test the response of two different populations of big sagebrush to drought stress. We found that these two populations exhibit distinct responses to drought stress.

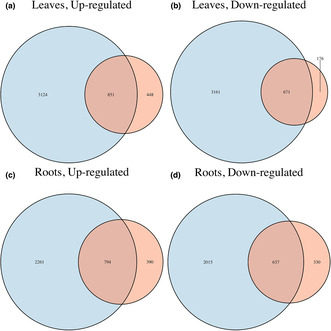

## 1 INTRODUCTION

Although significant progress has been made to model the trajectory of climate change (IPCC, 2021), little is known about the impact of climate change on natural ecosystems. Responses to extreme environmental conditions, such as extreme drought stress, have been well studied in model and crop systems, but there is a distinct knowledge gap for natural ecosystems and ecologically important non‐model species (Melton et al., 2022; Nadeau et al., 2017; Urban et al., 2016). Climate change is a major threat to biodiversity, but accurately predicting and creating practical solutions for the conservation of natural ecosystems has been difficult (Beever et al., 2016; Dawson, 2011; Nadeau et al., 2017; Nicotra et al., 2015; Urban et al., 2016). Applying an integrated framework focusing on determining species vulnerability to climate change based on (i) exposure (i.e., magnitude of climate change likely to be experienced by a species across its range), (ii) sensitivity (i.e., degree to which the performance, survival, and persistence of a species are affected by climate change), and (iii) adaptive capacity (i.e., potential for a species or populations to tolerate or adapt to climate change) will be essential for conservation efforts. The relative contributions of these components will lead to different management interventions to sustain populations in the face of climate change (Beever et al., 2016; Dawson, 2011; Nicotra et al., 2015).

There are three components of adaptive capacity (Murren et al., 2015; Ofori et al., 2017): (i) dispersal, (ii) local adaptation, and (iii) phenotypic plasticity. Dispersal allows organisms to move to regions with suitable habitats and promotes gene flow that increases genetic diversity, fitness, and evolutionary potential. Local adaptation is in situ microevolution that increases the fitness of local populations (reviewed in Blanquart et al., 2013). Finally, phenotypic plasticity is the ability of individuals to change their phenology, physiology, or morphology without changing their genetic makeup (Ofori et al., 2017). Phenotypic plasticity has been found to be correlated with climatic factors for plants and can mediate responses to climate change (Henn et al., 2018; Stotz et al., 2021).

One prominent effect of climate change will be more severe droughts across many regions (IPCC, 2021). Drought occurs due to a lack of precipitation and concomitant reduction in soil moisture, which has large‐scale impacts on plant species, communities, and ecosystems (Ault, 2020). Drought is a major cause of seedling mortality because seedlings are the most vulnerable stage of the plant life cycle, and the persistence and sustainability of plant communities are dependent on the survival and reproduction of individuals of species that form these communities (Leck et al., 2008). Thus, drought, and other aspects of the abiotic and biotic environment, exert variable selection on populations of species that lead to local adaptation (i.e., ecotypic differentiation among populations; Via & Lande, 1985). Consequently, plants that are locally adapted to specific environmental conditions will have high fitness while those conditions are maintained but will be more vulnerable to recruitment and reproductive failures as conditions change, such as those associated with climate change. Deciphering the mechanisms that produce the phenotypes contributing to seedling survival, recruitment, and successful reproduction is paramount to predict the impact of climate change on populations, and ultimately communities and ecosystems.

The severe and persistent 21st‐century megadrought in southwestern North America (SWNA) has been identified recently (Williams et al., 2020) based on hydrological modeling coupled with 1200‐year tree‐ring reconstruction of summer soil moisture. Williams et al. (2020) demonstrated that the 2000–2018 SWNA megadrought was the second driest 19‐year period since 800 CE, exceeded only by a megadrought in the late‐1500s, and this drought has now persisted into the 2020s (Williams et al., 2022). This recent extreme climate event was reported to be driven by natural variability superimposed on drying due to anthropogenic warming, with human activities accounting for 47% of the response (Williams et al., 2020). Although significant progress has been made in understanding the origin of recent megadroughts, we still lack the fundamental knowledge to predict how organisms will respond to these extreme climate events, which are likely to intensify in the future (Stott, 2016).

Research exploring the response pathways of plants to extreme climate events is especially important in ecosystems dominated by one or a few keystone plant species. The sagebrush steppe ecosystem of western North America is characterized by the keystone species *Artemisia tridentata* Nutt. (Asteraceae), and largely occurs within the region experiencing the SWNA megadrought. *Artemisia tridentata* provides shelter and food for many herbivores, including the endemic pygmy rabbit (*Brachylagus idahoensis*) and two species of sage‐grouse (*Centrocercus* spp.; Prevéy et al., 2010; Welch, 2005). The sagebrush steppe was once distributed across approximately one million km^2^ of western North America (Miller et al., 2011; Miller & Eddleman, 2001), but has since been destroyed and fragmented due to threats from invasive species (Prevéy et al., 2010), increased fire frequency and intensity (Shriver et al., 2019), habitat destruction (Thompson, 2007), and climate change (Richardson et al., 2017; Richardson & Chaney, 2018; Still & Richardson, 2015). These factors have resulted in a drastic decline in *A. tridentata* seedling recruitment, exacerbating population declines. Because of these threats, land managers have prioritized restoration efforts of sagebrush in these ecosystems with limited success (Arkle et al., 2014; Knutson et al., 2014); However, these efforts have not investigated how local adaptation and adaptive capacity may influence the survival and reproductive success of *A. tridentata*, and thus the success of restoration of this keystone species.

**FIGURE 1:**
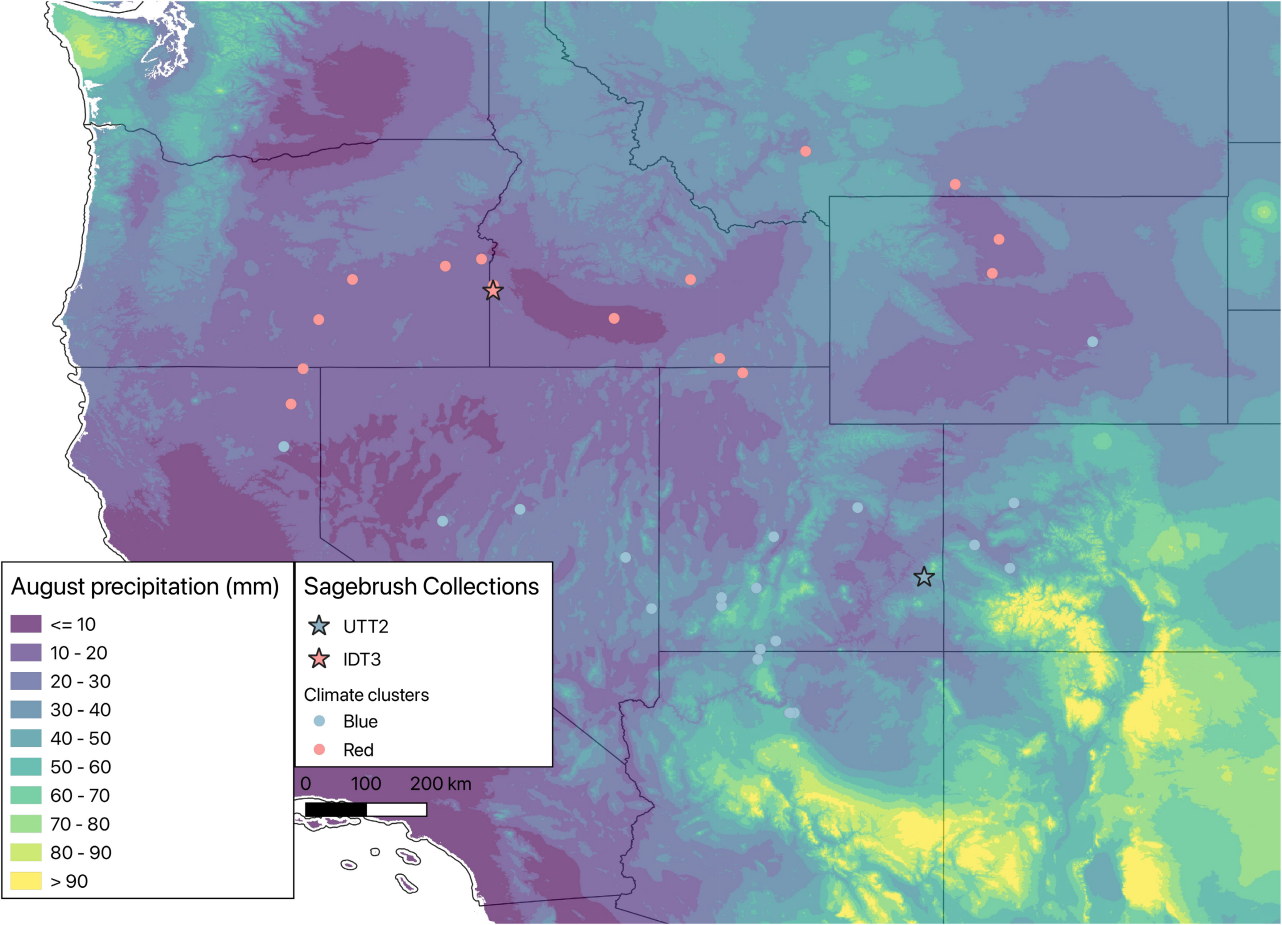
Map of collection sites for *Att* with the color of symbols corresponding to the PCA cluster identified using climatic data. The source sites for IDT3 (Red cluster) and UTT2 (Blue cluster) populations are designated by stars. Principal component one explained 80.3% of the variance, and principal component two explained 17.1% of the variance. Points are overlaid upon the average precipitation (mm) in the month of August from WorldClim v2.1, highlighting differences in the amount of rainfall received by each population at the arrival of the NAM.

*Artemisia tridentata* has a broad distribution, covering much of western North America, and includes a variety of climatic regimes (Figure 1). For example, populations of *A. tridentata* receive far less summer rain in the northwestern parts of the distribution and are generally under intense drought during the summer months, while southern parts of the distribution receive summer rain from the onset of the North American Monsoon (NAM; reviewed in Adams & Comrie, 1997). This can lead to instances of local adaptation to precipitation patterns that would otherwise be maladaptive in other parts of the *A. tridentata* distribution. Common garden experiments have shown that *A. tridentata* population of origin is a strong predictor of mortality when translocated to different areas across climatic gradients, particularly for minimum temperature (Chaney et al., 2017). This suggests that local adaptation to climate across sagebrush populations is an important factor in how individuals from various populations will respond to different winter climate regimes, though local adaptation may also drive responses to other climatic aspects such as summer drought conditions. Williams et al. (2020) found that there have been significant declines in soil moisture in the last two decades, with especially acute declines for the southern parts of the *A. tridentata* distribution, such as in Utah. Because of the lower precipitation levels in Idaho and higher survival of populations from Idaho in the Idaho common garden (Chaney et al., 2017), populations from Idaho may be more well‐adapted to extreme drought stress, making them ideal candidates to serve as seed sources for restoration efforts under conditions of predicted climate change. Research on exposure and sensitivity to climate change has been conducted (Chaney et al., 2017; Germino et al., 2018; Requena‐Mullor et al., 2019; Still & Richardson, 2015) though much less is known about the adaptive capacity of *A. tridentata*. Natural dispersal distance is limited for *A. tridentata* (Applestein et al., 2022), but local adaptation and phenotypic plasticity have been studied to a much lesser degree.

Little is known about the genetics and transcriptomics of *Artemisia tridentata* (Bajgain et al., 2011; Melton et al., 2021; Richardson et al., 2012), and this represents the first efforts to link phenotypic plasticity (i.e., variation between treatments within a population), population effects (i.e., variation between populations), and transcriptomic analyses for drought response in this species. This study aimed to answer three questions regarding the response and adaptive capacity of diploid *A. tridentata* subsp. *tridentata*, hereafter referred to as *Att*, seedlings: (i) what climate variables best explain local climate regimes that different *Att* populations experience?; (ii) are populations genetically distinct, therefore providing evidence for local adaptation?; (iii) how does local adaptation translate into an adaptive plastic response to drought, as determined from physiological and transcriptomic data? To answer these questions, a common garden genotype‐by‐environment (G × E) experiment was performed using two populations from climatically distinct areas. Following the drought G × E experiment, RNASeq and differentially expressed gene (DEG) analyses were performed. We hypothesize that plant populations originating from contrasting climatic conditions differ in their physiological responses and gene expression under imposed drought stress in a common garden and that these differences will be associated with photosynthetic performance.

## 2 MATERIALS AND METHODS

### 2.1 Climate analysis

Occurrence data were collected in October and November of 2020 when *Att* is largely in fruit. A total of 33 sites were sampled across the distribution of *Att* (Figure 1). Latitude and longitude were collected for 10 individuals at each site. Climate and elevation data from WorldClim (BioClim v2; https://worldclim.org/; Fick & Hijmans, 2017), as well as aridity index (AI) and potential evapotranspiration (PET) from CGIAR CSI (https://cgiarcsi.community/data/global‐aridity‐and‐pet‐database/, Zomer et al., 2007; Zomer et al., 2008), were downloaded at 30 arcsec resolution (~1 km^2^ at the equator). Each occurrence was then used to sample environmental data via the function *extract* from the “raster” package in R, which was then reduced to one sample per cell by filtering out samples with duplicate cell numbers. This left a total of 44 of 50 cells sampled across the sample areas of the geographic distribution of *Att* (Table S1). The sampled data were then used to perform a principal component analysis (PCA) using *prcomp* in base R v4.1.1 (R Core Team, 2021). The function *fviz_nbclust* from the R package “factoextra” v1.0.7 (Kassambara & Mundt, 2020) was used to determine the best number of clusters within the PCA results.

### 2.2 Genotype‐by‐environment experiment

In this study, photosynthesis and transpiration were estimated using chlorophyll fluorescence metrics and daily water loss, respectively. Based on the differing climatic regimes, we hypothesized that the abiotic conditions across the landscape have acted as selection drivers leading to local adaptation. To test this hypothesis, bi‐allelic Single Nucleotide Polymorphic (SNP) sites in linkage‐equilibrium were called from the transcriptomic data (see Gene Expression Analysis below) and used to conduct ancestral genotype structure analyses. These data will be key to predict the respective roles of genetics and phenotypic plasticity in drought response between the two model populations.

#### 2.2.1 Experimental design

The drought gene‐by‐environment (G × E) experiment was conducted in the Boise State University research greenhouse facility on *Att* seedlings grown from seed collected from two diploid (2*n* = 2x = 18) populations: one near the fringe of the Soda Megafire in southwestern Idaho (IDT3; 116.9641 W, 43.3366 N) and one from La Sal, Utah (UTT2; 109.3876 W 38.306 N; Table S1). The IDT3 population represents the Red cluster, whereas the UTT2 population represents the Blue cluster. Seeds from 10 maternal plants were collected from two distinct populations for the G × E experiment. Seeds from each maternal plant, hereafter referred to as a family, were sown on 10 July 2019 in the research greenhouse facility at Boise State University (Boise, Idaho, USA) and were grown for 7 months prior to starting the experiments. The growth time was set to ensure acclimation and minimize maternal effects as well as to ensure that leaves were of the right size to conduct physiological measurements. Due to the wind‐pollinated, highly outcrossing mating system of sagebrush, seeds from each maternal plant were considered to be half‐siblings. *Att* is a highly outbred species with few genomic resources available (Melton et al., 2021). While using in‐bred or clonal lines would be ideal for G × E experiments, the slow growth of *Att* precluded the development of sufficiently inbred lines. Thus, the family approach used here offers the best way to account for genetic variation within the experiment. Three seeds per maternal plant were sown directly into 983 cm^3^ Deepot™ (Stuewe & Sons, Inc.) containers with soil mix (1:1 v/v) composed of one part soil conditioners (one‐part volcanic cinder: two‐parts vermiculite: one‐part peat moss) and one‐part greenhouse potting mix (one‐part topsoil: one‐part compost). The soil mixture was designed by the USDA Forest Service to resemble a well‐drained dryland soil (Chaney et al., 2017). The soil conditioner in the soil mix allowed for sufficient drainage for the seedlings growing in the containers. The goal of using this soil mixture was to maintain a homogeneous soil mix, holding the environment constant, to observe differences in response based on genetic variation. The use of field‐collect soils could introduce a variety of pathogens unless sterilized, reducing similarity to the native untreated soils, and may also introduce further heterogeneity and sources of variation to the experiment. Drainage of the soil mix was verified by observing that soil at the top of the containers dried within 48 h following watering; drying also indicated that re‐watering was required to maintain the well‐watered experimental treatment (see *Drought G × E Experiment* below). Greenhouse temperature was maintained at 20°C (+/−2°C) and a 16/8 h day/night photoperiod was maintained throughout the experiment. Seeds and seedlings were watered on alternating days for optimum growth. Once seeds were sown, containers were randomly placed into racks that could accommodate 20 containers, and the positions of the racks in the greenhouse were randomized every 2 weeks to minimize possible greenhouse microclimate effects. Random thinning of seedlings occurred when more than one seed germinated to ensure that there was only one seedling per container. Mortality data were collected every 2 weeks, for approximately 7 months. Once mortality stabilized, plants were grown under optimum conditions for two more weeks before starting the drought G × E experiment to allow seedlings to acclimate to the greenhouse environment and reduce maternal environmental effects. Seedlings were grown in the greenhouse for a longer period of time than typically occurs under natural conditions, before the onset of summer drought. However, this approach was necessary to ensure that individual leaves and entire plants were large enough for physiological measurements.

#### 2.2.2 Drought G × E experiment

The objective of this experiment was to compare the physiological and transcriptomic responses of sagebrush seedlings from IDT3 and UTT2 at the onset of drought stress, in the absence of heat stress, and harvest seedlings for other analyses prior to death. The onset of drought stress was detected using leaf temperature, weight loss, and stomatal conductance (See *Physiological Measurements*). The drought experiment was terminated upon the majority of T2 seedlings exhibiting no water loss, estimated by weighing containers (Table S2) and measuring leaf temperature, suggesting that seedlings closed their stomata and entered into starvation to avoid death by hydraulic failure. Stomatal closure was confirmed using stomatal conductance (Table S3). The goal for the drought G × E experiment was to include six randomly selected seedlings per family, with three per treatment, and 10 families per population, resulting in 60 individuals per population. Therefore, a total of 120 individuals were to be included in this experiment. However, due to what appeared to be random mortality, some families had less than six individuals surviving; thus, the total sample size at the start of the experiment was 117 seedlings comprising 59 for IDT3 and 58 for UTT2. Seedlings were randomly allocated to treatments and trays within treatments. Trays were randomized each week to avoid possible greenhouse microclimate effects. The G × E experiment started on 24 February 2020 and lasted 16 days. Seedlings were randomly divided into treatments. T1 seedlings were watered every 2 days so that the soil mix was at field capacity, whereas T2 seedlings were well‐watered on day one, and then watering was withheld for 15 days. Withholding water caused the soil mix to dry from the top down, which mimics the typical soil drying pattern of sagebrush habitats during the late spring and summer, and simulates summer drought conditions (Hacke et al., 2000). Sagebrush seedlings were harvested at the end of the experiment, imaged (Nikon, model d5600), and stored at −80°C freezer for subsequent RNA analyses.

#### 2.2.3 Physiological measurements

The effectiveness of the imposed‐drought treatments was assessed by weighing all T2 containers, and a random subset of T1 containers, daily using an Ohaus Scout SPX8200 portable balance. These data were used as a proxy of water content of the soil mix, which determines the water availability for photosynthesis and plant growth. Normalized weight loss of containers through time, which was used as evidence to monitor and terminate the drought G × E experiment, was inferred using base R (R Core Team, 2021). Stomatal conductance was used to ascertain the timing of stomatal closure. Stomatal closure is considered a sign of drought stress as leaves have reached negative carbon balances because of severe soil water deficits (Pirasteh‐Anosheh et al., 2016). This was used as additional evidence to terminate the drought treatment. Stomatal conductance (mmol m^−2^ s^−1^) was measured on a random subset of T1 (*n* = 5) and T2 (*n* = 30) seedlings using a model SC‐1 leaf porometer (Decagon Devices, Inc.; Table S3). Instrument calibration was conducted before each set of measurements at the end of treatment period according to the manufacturer's guidelines. Leaves of sagebrush seedlings are relatively small and have stomata on both sides of the blade (Downs & Black, 1999). For each measurement, three persistent leaves were inserted in the porometer to fully cover the sensor aperture. This was used as additional evidence to terminate the drought treatment. Upon termination of the drought G × E experiment, photosynthesis performance of T1 and T2 seedlings was determined based on leaf chlorophyll fluorescence and leaf temperature (°C) using a MultiSpeq v2 PAM fluorometer (PhotosynQ Inc.; Table S4). Photosynthetic electron transport relies on a sufficient amount of water to produce chemical energy (ATP and NADPH), which is then used for carbon fixation and the production of sugar molecules that sustain plant growth (Murchie & Lawson, 2013). The effect of drought on the photosynthetic performance of sagebrush seedlings was assessed in light‐adapted leaves using the MultiSpeq v2 a PAM fluorometer following the Photosynthesis RIDES protocol (Kuhlgert et al., 2016) to collect Phi2 (Fq′/Fm′; the operating efficiency of Photosystem II photochemistry) and PhiNPQ (ratio of incoming light going toward non‐photochemical quenching). For each measure, a single mature leaf was inserted into the device cuvette so that the entire leaf was within the light guide and this procedure was repeated on two different leaves. The order that the plants were measured was randomized daily. Due to their small size, some seedlings were not suitable for chlorophyll fluorescence and stomatal conductance measurements and were therefore not included in the analyses (Tables S3 and S4).

#### 2.2.4 Physiological statistical analyses

Statistical analyses were conducted based on leaf temperature, Phi2, and PhiNPQ physiological data gathered from two individuals per family per treatment for a total of 10 families per population. To account for non‐independence in individuals within a family, measurements from the two individuals per family were averaged and used as input for boxplots to visualize trends and generalized linear model (GLM) analyses. Thus, each family represents a replication with 10 replications per treatment and population. Population effects within treatments and phenotypic plasticity within population between treatments were assessed based on physiological metrics using GLMs as implemented in the *glm* function in stats R v4.1.1 (R Core Team, 2021). The boxplots were generated using the *boxplot* function in base R v4.1.1 (R Core Team, 2021).

### 2.3 Gene expression analysis

#### 2.3.1 Tissue sampling and RNA extractions

Approximately 0.1 g of frozen leaf and root tissue per individual were sampled for RNA extractions. Samples were placed into five 96‐well plates and shipped to the Center for Genome Research and Biocomputing laboratory at Oregon State University for RNA extraction. RNA extraction was performed using Omega Bio‐Tek (Norcross, GA, USA) E‐Z 96 plant RNA extraction kits (item number R1027‐02). A total of 152 samples comprising leaf and root RNA extractions 38 seedlings of each population (19 T1 and 19 T2 seedlings for IDT3, 18 T1 and 20 T2 seedlings for UTT2), were selected for shipment to HudsonAlpha Genomics Institute (Huntsville, AL, USA) for sequencing. Library preparation for 2 × 150 bp paired‐end read sequencing was performed. Sequencing was performed on 3 lanes of an S4 Illumina NovaSeq 6000 flowcell (Illumina).

#### 2.3.2 Reference transcriptome assembly and read mapping

Reads were assessed for quality and trimmed using Trimmomattic v0.39 (Bolger et al., 2014). A reference transcriptome was assembled from cleaned readsets of one well‐watered and one drought‐stressed set of leaf and root samples from IDT3 (seedlings 89 (T2) and 171 (T1)). These samples were chosen as they were the largest readsets representing the two treatments. The reference transcriptome was assembled using Trinity v2.12.0 (Grabherr et al., 2011). The reference transcriptome was then assessed for completeness using BUSCO v5.2.2 using both eukaryota_odb10 and eudicots_odb10 databases (Simão et al., 2015). The reference transcriptome was then annotated using the Trinotate v3.2.2 (Bryant et al., 2017) pipeline. Cleaned reads for all other samples were then mapped to the reference transcriptome using Bowtie2 v2.4.4 (Langmead & Salzberg, 2012) using the—very‐sensitive flag. Read counts were then calculated using the samtools v1.14 (Danecek et al., 2021) *idxstats* tool.

#### 2.3.3 Ancestral genotype structure analyses

To determine the effect of local adaptation on the physiological and transcriptomic performance of seedlings under drought treatment, ancestry analyses were conducted on the transcriptomic data. Such analyses are also important since we are conducting our comparative analyses at the population level and large genetic variation within populations could artificially inflate phenotypic plasticity. Principal component and admixture analyses were performed on all samples for which both root and leaf transcriptomes were included in the differential expression analyses. Root and leaf Binary Alignment Map (BAM) files were merged using the “samtools” *merge* tool (Danecek et al., 2021). Variants were called using bcftools v1.14 *mpileup* (Danecek et al., 2021), with a random seed of 1234. Only variants with a minimum mapping quality score of 20 and a base calling score of 30 were called. Called variants were indexed, then filtered to remove any multi‐allelic SNP or variants within five bases of an indel. The final SNPs were then filtered to a read depth that fell above the 5th or below the 95th quantiles using the “vcfR” v1.12.0 R package (Knaus & Grünwald, 2017). Any remaining SNPs were filtered to a read depth of 4. Samples missing greater than 90% of the remaining data and SNPs missing from greater than 50% of samples were excluded from analyses. The *snpgdsLDpruning* function of the “SNPRelate” v1.26.0 R package (Zheng et al., 2012) was used to identify variants in linkage disequilibrium with a threshold of 0.2, which were removed prior to the admixture analysis. For the principal component analysis, the *snpgdsPCA* function of the “SNPRelate” R package was used. Finally, the “LEA” v3.4.0 R package (Frichot & François, 2015) was used to perform the admixture analysis. *k*‐values of one to 10 were tested, with a total of 10 repetitions with 1000 iterations and the lowest cross‐entropy criterion for selecting the best *k‐*value. The *basic. stats* function of the “hierfstat” v0.5–11 R package (Goudet, 2005) was used to calculate Fst for the SNPs used in the previous analyses.

#### 2.3.4 Differential expression analyses

Differential expression (DE) analyses were performed using the R package “edgeR” v3.34 (Robinson et al., 2010). The design matrix was set up so that each combination of tissue, population, and treatment was represented as its own group. Transcripts were filtered by expression levels, informed by the design matrix, using the *filterByExpr* function. Read count normalization factors were estimated using the *calcNormFactors* function with the “Trimmed Mean of *M*‐values” normalization (TMM) method. Dispersion was calculated using the *estimateDisp* function with the “robust” flag set to “TRUE.” Preliminary evaluation of expression patterns was performed using the *plotMDS* function (Figure S1a,b).

Contrasts were defined to allow for comparisons between treatments within a population and tissue (e.g., T2 IDT3 leaf—T1 IDT3 leaf). A GLM was fitted to the expression data using the *glmQLFit* function. DE tests for each set of contrasts were performed using the *glmQLFTest* function. The *decideTetstsDGE* function was then used to identify DEGs using the Benjamini‐Hochberg Procedure (BH method; Benjamini & Hochberg, 1995) of *p*‐value adjustment and a *p*‐value threshold of .05.

#### 2.3.5 GO category enrichment analysis

DEGs were analyzed for Gene Ontology (GO) category enrichment using the Gene Ontology GO Enrichment Analysis web interface (http://geneontology.org/; GO Ontology database DOI: 10.5281/zenodo.6399963). List of gene names for transcripts identified by Trinotate was used as input for analysis. *Arabidopsis thaliana* was used as the reference for GO Enrichment analysis. The Fisher's Exact test method was applied during analyses. *p*‐value correction was performed using the “False Discovery Rate” method using the BH method.

## 3 RESULTS

### 3.1 Climate analysis

PET and elevation were found to contribute the most to the top two principal components, with the BioClim layers contributing relatively little to these principal components. Principal component one explained 80.3% of the variance, and principal component two explained 17.1% of the variance. Results of the PCA and clustering analysis show that the distribution of *Att* comprises two distinct clusters of environmental niche space with a distinct southwest/northeast divide. IDT3 falls within the Red Northwest cluster, while UTT2 falls within the Blue Southeast cluster (Figure 1).

### 3.2 Drought G × E experiment

G × E experiment treatments were terminated after 15 days, once water loss had decreased and leaf temperature had increased. The effectiveness of the drought treatment was verified by the decline in loss of weight from evapotranspiration (Figure S2; Table S2). Within 1 h of terminating the experiment, T1 seedlings had a mean stomatal conductance of 214.44 mmol m^−2^ s^−1^ (SD = 72.47 mmol m^−2^ s^−1^) and T2 seedlings had a mean stomatal conductance of 100.58 mmol m^−2^ s^−1^ (SD = 94.50 mmol m^−2^ s^−1^).

### 3.3 Physiological statistical analyses

**FIGURE 2:**
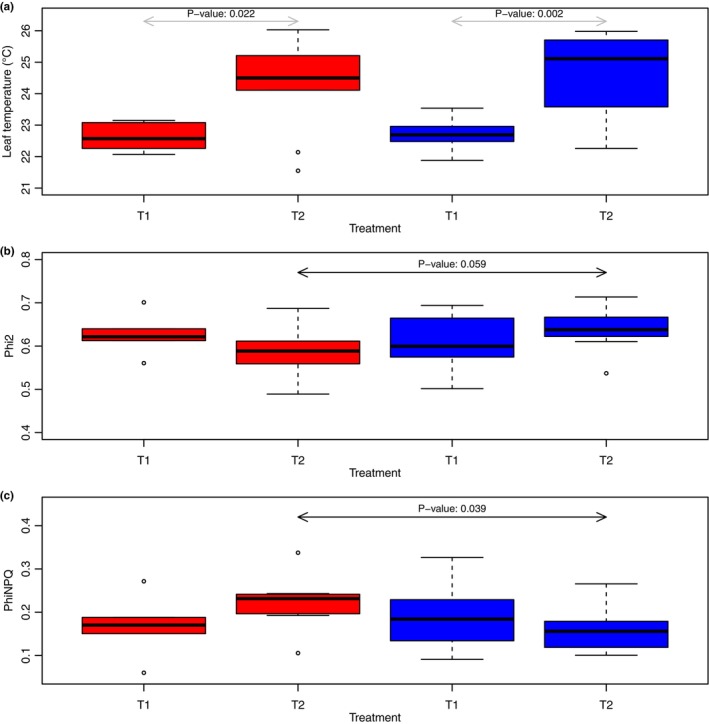
Comparisons of leaf temperature (a) Phi2 (b), and PhiNPQ (c) parameters between treatments and populations. Plants from both populations experienced statistically significant (UTT2 *p*‐value = .022; IDT3 *p*‐value = .002) increases in leaf temperatures between treatments (a), denoted by the arrows above the boxplots. A marginal population effect (*p*‐value = .059) was found for Phi2 (b) and a statistically significant population effect (*p*‐value = .039) was found for PhiNPQ (c), with UTT2 maintaining higher photosynthetic efficiency under drought stress than IDT3 per these metrics. Legend: T1: well‐watered treatment; T2: drought treatment; red boxplots are for IDT3 seedlings, whereas blue boxplots are for UTT2 seedlings; arrows represent statistically significant results based on GLM analyses with their associated *p*‐values, with black arrows representing population effects with treatment and gray arrows representing phenotypic plasticity within the population between treatments.

Seedlings from both populations exhibited statistically significant phenotypic plasticity, with increases in leaf temperature in their respective T2 treatment groups relative to T1 groups (IDT3 *p*‐value = .022; UTT2 *p*‐value = .002; Figure 2a). There was a marginal population effect for Phi2, with T2 UTT2 seedlings maintaining higher Phi2 than T2 IDT3 seedlings (*p*‐value = .059; Figure 2b). There was a statistically significant population effect for PhiNPQ, with IDT3 T2 seedlings having higher PhiNPQ values than those of UTT2 T2 (*P*‐value = .039; Figure 2c) seedlings.

### 3.4 RNASeq, reference transcriptome assembly, and read mapping

A total of 152 leaf and root tissue samples passed quality control for RNASeq. These samples represent 76 seedlings used in the drought G × E experiment. Read counts of datasets used in subsequent analyses ranged from 1,008,911 read pairs to 189,184,514 read pairs (Table S5).

The reference transcriptome contained 938,307 transcripts representing 338,820 Trinity/Trinotate gene groups. BUSCO analysis found that 93.3% (238 of 255) eukaryota_odb10 BUSCOs and 78.2% (1817 of 2326) eudicots_odb10 databases BUSCOS were completely assembled, with only 0.8% (*n* = 2) and 11.6% (*n* = 272) missing from the assembly for eukaryota_odb10 and eudicots_odb10 BUSCOs, respectively. Trinotate recovered putative transcript identities for 36.35% (341,036 of 938,307) of transcripts.

A total of 122 RNASeq read sets comprising data from 72 seedlings (66 T1 and 56 T2 tissue samples) contained more than one million reads and were used for read mapping and DE analysis. The mean percent mapping was 58.06%, with a range of 12.65–78.81% and a standard deviation of ±12.36% (Table S6).

### 3.5 Ancestral genotype structure analyses

**FIGURE 3:**
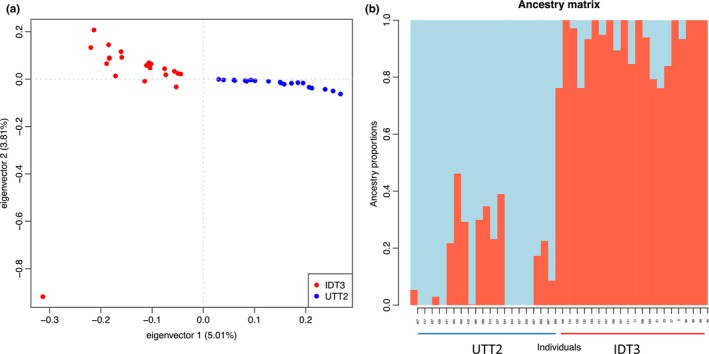
Visualization of principal component analysis (a) and bar plot (b) showing admixture of ancestral genotypes present in the IDT3 (red) and UTT2 (blue) populations. Each population formed distinct clusters along a gradient on PC1. For the admixture analysis, a *k*‐value of two was selected based on the lowest cross‐entropy score criterion (cross‐entropy = 0.4525813). While admixture was found, all individuals were predicted to be members of their respective populations.

A total of 50 seedlings had both leaf and root RNA data meet the criteria for inclusion in the genotyping analyses. A total of 107,777 SNPs from 41 samples with 31.64% missing data remained after SNP processing. Principal components one and two comprised 5.01% and 3.81% of variance, respectively. Two distinct population‐level clusters were identified using the PCA (Figure 3a). A *k*‐value of two was selected for admixture analysis based on the cross‐entropy scores (Figure S3; cross‐entropy score of the best‐performing run was 0.4525813). Admixture analysis showed low levels of admixture between the two ancestral genotypes (Figure 3b). Genetic structure largely was based on population, with no member of a population being predicted to be a member of the other. Minimum *Q* scores for primary ancestral genotype were 0.761 and 0.538 for IDT3 and UTT2, respectively. Fst was calculated to be 0.0517.

### 3.6 DEG analysis

Preliminary evaluation of expression patterns using the *plotMDS* function revealed one outlier sample and was thus removed (Figure S1a,b). This left 121 tissue samples from 71 seedlings, comprising 65 and 56 tissue samples from T1 and T2 treatment groups, respectively, remaining for further analysis. A total of 80,194 differentially expressed transcripts, representing 15,775 trinotate gene groups, were identified across comparisons of treatment and population (Table S7).

**FIGURE 4:**
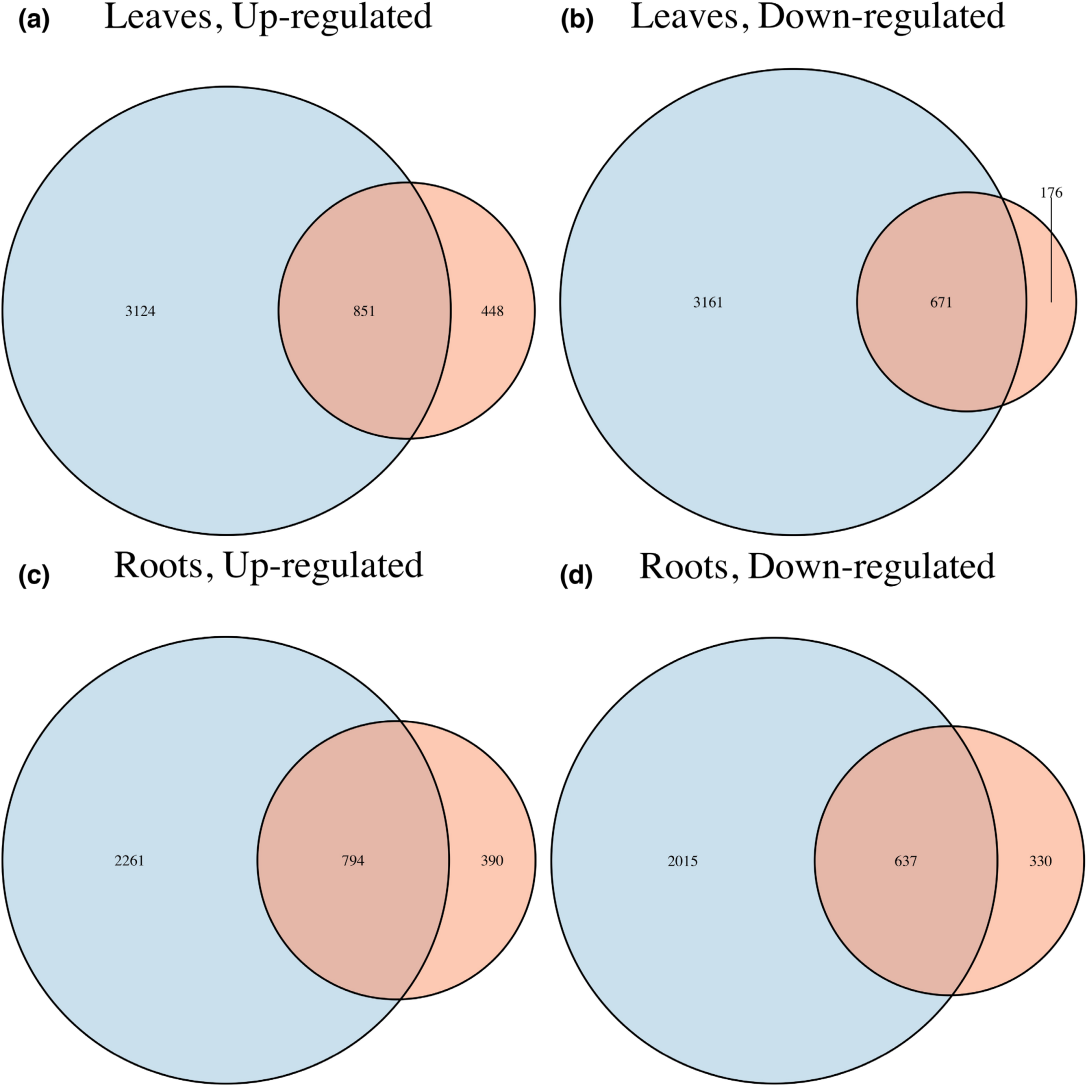
Venn diagram showing overlap of up‐ or down‐regulated DEGs identified in IDT3 (red) and UTT2 (blue) tissues and annotated by Trinotate. The size of the circle is proportional to the number of up‐ or down‐regulated DEGs from T1 (well‐watered) to T2 (drought‐stressed) for each tissue type. For all comparisons, UTT2 samples had far more up‐ and down‐regulated DEGs, with only about two‐thirds of DEGs identified in IDT3 samples being shared with UTT2 samples. For example, in (a), UTT2 seedlings had 3124 uniquely up‐regulated genes in leaf tissue versus 448 uniquely up‐regulated genes in IDT3 seedling leaf tissue, with only 851 shared up‐regulated DEGs.

The UTT2 population was found to have more unique DEGs within each comparison relative to the IDT3 population (Figure 4). Within leaf tissue, UTT2 had 3124 unique up‐regulated and 3161 unique down‐regulated DEGs compared to 448 unique up‐ and 176 unique down‐regulated DEGs in IDT3. Within root tissue, UTT2 had 2261 unique up‐regulated and 2015 unique down‐regulated DEGs compared to 390 unique up‐ and 330 unique down‐regulated DEGs in IDT3. A total of 851 up‐regulated and 671 down‐regulated leaf DEGs were shared between IDT3 and UTT2. A total of 704 up‐regulated and 637 down‐regulated root DEGs were shared between IDT3 and UTT2 (Figure 4).

### 3.7 GO enrichment

GO enrichment analyses for up‐regulated DEGs shared between populations in leaf tissue reveal that genes that function in biochemical pathways, particularly ATP and ADP transport, protein refolding (e.g., de novo, post‐translation, and response to improperly or unfolded proteins), and responses to abiotic (e.g., temperature, oxygen, hydrogen peroxide, metal ions, water, and ABA) and biotic stresses (e.g., fungal and bacterial) are associated with drought‐stress response in both IDT3 and UTT2 populations (Table S8). Genes that function in cell division and reproductive processes (e.g., meiotic and cytokinetic processes, DNA replication and repair, organ/tissue morpho‐ and histogenesis) were down‐regulated in both leaf and root tissue for both populations. Shared DEGs for root tissue comprise similar GO categories as those in leaf tissue (e.g., protein refolding, response to heat and oxidative stresses), but also include phytochemical (e.g., ethylene, alkene, inositol, olefin, lignin, salicylic acid, oxylipin, lipids) processing, responses to UV light, and phosphate starvation.

The IDT3 population had fewer GO categories enriched in their unique DEGs than UTT2 (Table S8). A total of 16 and 347 enriched Biological Process GO categories in unique up‐regulated leaf tissue DEGs were found for IDT3 and UTT2, respectively. A total of 21 and 534 enriched Biological Process GO categories in unique down‐regulated leaf tissue DEGs were found for IDT3 and UTT2, respectively. A total of 28 and 357 enriched Biological Process GO categories in unique up‐regulated root tissue DEGs were found for IDT3 and UTT2, respectively. A total of 24 and 418 enriched Biological Process GO categories in unique down‐regulated root tissue DEGs were found for IDT3 and UTT2, respectively.

## 4 DISCUSSION

Overall, this research shows these populations of *Att* exhibit different drought responses in a controlled G × E experiment. UTT2 seedlings maintain higher photosynthetic rates and efficiency under imposed drought stress than those of IDT3, as indicated by higher Phi2 and lower PhiNPQ scores (Figure 2). The two populations also exhibit distinct transcriptomic response pathways, with UTT2 seedlings exhibiting far greater transcriptomic responses than those of IDT3 (Figure 4). These results are counter to what may be expected given differences in respective climates (i.e., IDT3 seedlings would perform better under imposed drought conditions than those of UTT2 due to longer natural droughts in Idaho versus Utah). While the survival of *Artemisia tridentata* from Utah is lower than of Idaho in common gardens based in Idaho (Chaney et al., 2017), it was found that differences in survival within the experiment were best explained by minimum temperature. The previous common garden experiment included the three major subspecies of *A. tridentata* and diploid and tetraploid cytotypes of each subspecies versus the narrower focus of the G × E experiment described here. Another key difference is that previous common garden experiments were performed in outdoor plots versus a climate‐controlled greenhouse. Performing the drought G × E in the greenhouse allows for a fine‐scale focus on the response of seedlings to only one environmental variable. Our work also focused solely on short‐term drought response, rather than long‐term survivability. The differences in scale (i.e., multiple subspecies and cytotypes versus only diploid *A. tridentata* subsp. *tridentata*, natural climate common garden versus climate‐controlled greenhouse, multi‐year versus short‐term treatment) may explain differences between previous common garden results and the results described here.

### 4.1 Local adaptation to different climatic conditions

Our model populations were found to occupy distinct climate regimes, per the PCA, largely driven by PET (Figure 1). Given the differences in precipitation regimes due to NAM and general climatic conditions (Figure 1), local adaptation could drive differential response strategies across populations of *Att*. Two widespread desert shrub species, *Ambrosia dumosa* and *Larrea tridentata*, also occur over a broad range of microclimates, including temperature and precipitation, gradients influenced by elevation and the NAM (Custer et al., 2022). These two species also exhibit specialization in their respective local environments, though through different mechanisms.

Plants of both the IDT3 and UTT2 populations experienced an increase in leaf temperature, indicating that drought stress was achieved in the T2 treatment group for both populations. While both T2 treatment groups experienced drought stress, different population responses at both phenotypic and transcriptomic expressions were identified. In response to withholding water, seedlings from both populations showed an increase in leaf temperature, which most likely reflects a decrease in stomatal conductance due to experimentally imposed drought stress (Figure 2a). UTT2 seedlings maintained higher Phi2 under drought stress than IDT3 seedlings (Figure 2b). IDT3 seedlings dissipated a larger proportion of the light absorbed by photosystem II in non‐photochemical processes, indicated by the higher NPQ of IDT3 plants (Figure 2c). These metrics indicate that seedlings of the UTT2 population maintain higher photosynthetic efficiency under drought‐stress conditions, compared to seedlings of the IDT3 population.

The DEGs shared between the drought‐stress response pathways of IDT3 and UTT2 may be of particular interest to researchers focused on adaptations to drought and may represent a universal component of drought‐stress response pathways. Osmoprotectant genes commonly identified in broad‐scale transcriptomic drought response experiments (e.g., cytochrome P450, heat shock protein, late embryogenesis abundant proteins; reviewed in Augustine, 2016; Melton et al., 2022) were also identified among the shared up‐regulated DEGs. GO enrichment analyses for shared up‐regulated DEGs in leaf tissue reveal that genes that function in biochemical, particularly ATP and ADP transport, protein refolding, and responses to abiotic and biotic stresses are important to drought‐stress response in both IDT3 and UTT2 populations. Genes that function in cell division and reproductive processes were down‐regulated in both leaf and root tissue for both populations. Shared DEGs for root tissue comprise similar GO categories as those in leaf tissue, but also include phytochemical processing, responses to UV light, and phosphate starvation. The experimentally imposed drought increased the expression of stress response and proteome maintenance proteins, such as those from genes whose products function in protein folding processes based on the GO categories of DEGs. Both populations also responded to drought stress by reducing processes of cell division and DNA replication, indicating that both populations reduce expression of genes in growth and reproduction pathways, per GO category enrichment analyses (Table S8), most likely to conserve resources while experiencing drought conditions.

DEG analyses revealed population effects within treatment groups and tissues, indicating that the two populations have evolved contrasting regulation mechanisms and metabolic pathways to respond to drought stress (Figure 4a–d). Changes in gene expression, indicative of plastic responses, occurred in seedlings of both populations. The IDT3 samples were far more canalized (i.e., limited plastic response) in their transcriptomic responses to drought stress and had far fewer DEGs under drought‐stress, compared to samples of UTT2 (e.g., 1299 vs. 3975 up‐regulated DEGs in drought‐stress leaf tissue per population, respectively; Figure 4a–d). There is also little overlap between the DEGs between each set, with only about two‐thirds of IDT3 DEGs being shared with UTT2 (Figure 4a–d). In addition, UTT2 up‐regulated more genes involved in water transport and osmotic adjustment (e.g., *CLC*) than IDT3. The latter process allows plants to extract more water from drying soils and, via turgor maintenance, prolong root growth and photosynthesis (Turner, 2018). These results suggest that UTT2 plants have a plastic response and shift their metabolism to adjust to environmental conditions, whereas the metabolism of the IDT3 genotype largely lacks plastic response, and therefore appears to be more locally adapted to the specific climatic conditions.

The use of clones or inbred lines is ideal for G × E experiments as they reduce the effects of genetic variation on phenotypic expression. To date, in‐bred or clonal lines have not been practical for research in *Att* due to its slow‐growing nature. Given these obstacles, the half‐sib approach of replication is the best method to have some control over genetic variation among the plants of this experiment. While the admixture analysis supports two ancestral genotypes for our model populations, the Fst of 0.0517 indicates that there is low genetic differentiation among samples of the populations used in these analyses. This greatly decreases the likelihood of obtaining biased or misleading results due to the out‐crossing nature of *Att* or lack of genetic clones used in the experiment. The inferred admixture of ancestral genotypes is likely the result of biogeographic processes earlier within the diversification of *Att*, rather than recent cross‐breeding, due to the large geographic separation of the two populations. *Artemisia* likely arrived in North America from Asia via the Bering Land Bridge by 12 Mya, leading to the sagebrush steppe becoming a dominant ecosystem in western North America (Davis & Ellis, 2010). The North American clade of *Artemisia* likely began to diversify from its Asian sister clade approximately 10.8 Mya (Garcia et al., 2011; Sanz et al., 2011).

### 4.2 Transcriptomic plasticity drives differential drought responses

While both populations were under drought‐induced stress as indicated by changes in leaf temperature and conductances, clear differences in phenotypic and transcriptomic responses were identified between the IDT3 and UTT2 populations. UTT2 appeared to be less phenotypically plastic and able to better manage responses to drought stress. For example, IDT3 plants increased PhiNPQ under drought‐stress conditions, while UTT2 did not experience statistically significant plasticity for PhiNPQ. This indicates that IDT3 plants became less photosynthetically efficient than those of UTT2, dissipating a larger amount of incoming light under drought conditions. These differences in phenotypic plasticity and responses may be, in part, due to differences in the transcriptomic responses of individuals of each population. UTT2 individuals have much broader transcriptomic responses than IDT3 individuals. Within the sets of DEGs unique to UTT2 were many transcriptional and chromatin conformation regulation genes. These regulatory shifts may cause different chromatin conformations affecting transcription of key genes leading to the observed differences in phenotypic plasticity (Jaligot & Rival, 2015; Moler et al., 2018), and potentially increasing their overall fitness under drought stress.

Epigenomics and regulation of RNA silencing genes also appear to underpin the differential responses of seedlings of each population. Using GO enrichment analyses (Mi et al., 2013), we demonstrated that genes that function in epigenomic and RNA silencing pathways (e.g., nucleosome remodeling, DNA methylation, and RNA silencing) are greatly enriched in the down‐regulated DEG set in UTT2 tissues, but not in IDT3 tissues. Differences in nucleosome remodeling and de novo DNA methylation processes may have led to novel epigenomic states in UTT2 plants in response to drought, altering patterns of gene expression (Figure 4). Additionally, the expression of RNA silencing genes is also down‐regulated in UTT2 under drought, including AGO genes that code for the main component of the RNA‐induced silencing complex binding to short guide RNAs (e.g., miRNA), which results in cleaving mRNAs and repressing gene expression.

Differences in stress response due to local adaptation have been found in a variety of contexts (e.g., drought, heat, and cold stress). Populations of *Forsythia suspensa* (Oleaceae) have been found to vary in number of DEGs under drought conditions, but these differences do not appear to be due to differences in GO category enrichment. The DEGs of all populations studied largely comprise photosynthesis, oxidation–reduction, and cell membrane component‐related genes (Li et al., 2021). Populations of *Eucalyptus grandis* (Myrtaceae) have been found to vary in the expression of Heat Shock Proteins, but studies in this species are limited to previously identified heat‐stress responsive genes or gene families (Maher et al., 2019; Nguyen et al., 2017).

## 5 CONCLUSIONS

Overall, these results suggest that the UTT2 population responds to reduced soil moisture through an induced drought response and may be more well‐adapted to rapidly respond to drought stress compared to the IDT3 population. IDT3 exhibits a constitutive drought priming strategy. Such adaptations mirror contrasting environmental conditions in each region, with the Idaho location experiencing long summer droughts each year that are not ended by the NAM. Apart from differences in the scale of transcriptomic changes, there were also differences between the most up‐ and down‐regulated genes. Because many of these genes may have more than one function and can be involved in responses other than drought (Tang & Bassham, 2022; Zhou et al., 2011), it is difficult to infer the significance of these differences in gene expression in the plant response to drought exclusively.

This work has important implications for the conservation of *A. tridentata*. There are large efforts to reseed areas of sagebrush steppe that have been damaged by fire or anthropogenic activity. The success of these efforts is often low, with no recruitment for many reseeded areas in a given year (Lysne & Pellant, 2004). This study lays a foundation to understand the local adaptation of *Att* to drought stress. This information could be used to refine seed transfer and screen for drought‐adapted genotypes, ensuring better chances of sagebrush establishment and long‐term resiliency. Future research including broader ranges of phenotypes, a narrower set of genes and SNPs, and more representative populations will allow for deeper investigations into the mechanisms of adaptation among populations of this keystone shrub species.

## CONFLICT OF INTEREST STATEMENT

The authors have no competing interests to declare.

## Supporting information


Table S1.

Table S2.

Table S3.

Table S4.

Table S5.

Table S6.

Table S7.

Table S8.

Figure S1.
Figure S2.
Figure S3.

## Data Availability

The data that support the findings of this study are openly available from NCBI Sequence Read Archive (SRA) database within BioProject accession PRJNA934360 and the University of Idaho open data repository at https://doi.org/10.7923/H898‐2606. Scripts to support data processing and analyses described in this study are available at https://github.com/aemelton/Drought_GxE_PEI_2023.
