# Supplementary material for: A genotype × environment experiment reveals contrasting response strategies to drought between populations of a keystone species (Artemisia tridentata; Asteraceae)
Source: Plant Environ Interact. 2023 Jul 24;4(4):201–14. doi: 10.1002/pei3.10119 (PMC10423975; doi:10.1002/pei3.10119)
Supplement: Supplementary file 9 — Figure S1. Figure S2. Figure S3. [file PEI3-4-201-s006.docx]

Figure S1. Multi-dimensional scaling (MDS) analysis of gene expression data using the *plotMDS* function of the ‘edgeR’ R package. One outlier sample was identified and removed. Samples are separated by tissue on the X-axis, with leaf tissue samples being positive and root tissue samples being negative, and by treatment on the Y-axis, with T1 (well-watered) samples generally having a greater Y-axis value than T2 (drought-stressed) samples.

Figure S2. Normalized weight loss per container per day during the drought treatment.

Figure S3. Cross-entropy scores of k 1-10 for admixture analysis. Results of this analysis support a *k*-value of two (cross-entropy = 0.4525813).
